# Effect of Mouthwashes on the Mechanical Properties and Color Stability of Composite Material

**DOI:** 10.3390/ma19071304

**Published:** 2026-03-25

**Authors:** Saja Adeeb, Artur Kriger, Jarosław Żmudzki, Jacek Kasperski, Grzegorz Chladek

**Affiliations:** 1 Adeeb Clinic, 73/1 Legionów Polskich Str., 41-300 Dąbrowa Górnicza, Poland; saja.adeeb@onet.pl; 2 Department of Dental Prosthetics, Division of Medical Sciences in Zabrze, Medical University of Silesia in Katowice, 15 Poniatowskiego Street, 40-055 Katowice, Poland; artur.kriger@sum.edu.pl (A.K.); protstom@sum.edu.pl (J.K.); 3 Department of Engineering Materials and Biomaterials, Faculty of Mechanical Engineering, Silesian University of Technology, 18a Konarskiego Str., 41-100 Gliwice, Poland; jaroslaw.zmudzki@polsl.pl; 4 Materials Research Laboratory, Faculty of Mechanical Engineering, Silesian University of Technology, 18a Konarskiego Str., 41-100 Gliwice, Poland

**Keywords:** dental composites, mechanical properties, mouthwashes, aesthetic properties

## Abstract

Interactions between the chemical compounds contained in mouthwashes and the components of dental composites may significantly influence their functional properties. The study investigated the effect of mouthwashes on the mechanical properties and color stability of a restorative nanocomposite. Twelve mouthwashes characterized by different chemical compositions were selected and distilled water was used as a control. Composite specimens were conditioned for 24 h, 48 h, and 72 h. Changes in microhardness (HV), compressive strength (CS) and diametral tensile strength (DTS) were evaluated. In addition, color changes were analyzed using spectrophotometric measurements. The results demonstrated a significant decrease in both HV and CS after exposure to mouthwashes, depending on their composition and immersion time. DTS values remained stable. Color changes were statistically significant; however, the values observed remained within clinically acceptable limits from an esthetic standpoint. The loss of mechanical properties was generally most pronounced during the initial period of contact between the mouthwashes and the tested material, which should be considered as unfavorable. The influence of ethanol molecules on mechanical properties was not stronger than that of many ethanol-free solutions. The frequently suggested correlation between changes in composite material hardness and the pH values of mouthwashes was not confirmed.

## 1. Introduction

The impact of pathogenic microorganisms on dental and oral health is as old as the human species itself. Evidence indicates that hominins suffered from dental caries more than one million years ago [1], and archaeological findings of attempted caries treatment, in the form of drilling marks, date back to the Neolithic period [2,3,4,5]. Long before the nature of dental plaque and its relationship to oral diseases had been understood, humans recognized the importance of oral hygiene. Although, due to the perishable nature of materials, there is a lack of direct archaeological evidence for the use of chemotherapeutic agents for oral hygiene purposes, the earliest written records from China describing mouth rinsing with children’s urine for medicinal purposes date to approximately 2700 BC; later sources include descriptions of mouth rinses in the works of Pliny, Hippocrates, and in the Talmud [6,7]. Despite the passage of millennia and the tremendous advances in dentistry over recent decades, the effective prevention and treatment of dental caries, as well as the maintenance of proper oral hygiene, remain ongoing challenges.

One of the landmark developments in the management of the consequences of dental caries was the introduction of composite materials, which currently form the foundation of modern conservative and aesthetic restorative dentistry and are widely used for the restoration of hard dental tissues [8]. Their widespread adoption is attributable to a favorable combination of mechanical properties, aesthetic performance, and the ability to achieve adhesive bonding to tooth tissues [9,10,11,12], which is achieved through the development of complex combinations of cross-linking monomers, fillers with diverse chemical compositions, morphologies, and particle sizes, coupling agents, and photoinitiating systems [13]. At the same time, these materials are continuously exposed to the oral environment, including hygiene agents such as mouthwashes, which are multi-component formulations containing chemical compounds whose molecules can interact with the composite.

Modern mouthwashes are defined as solutions used as auxiliary preparations to prevent dental caries and periodontal diseases. They are primarily used for the chemical control of dental plaque, acquired pellicle, tartar, as well as for tooth whitening, halitosis prevention, enamel remineralization, and reduction of dentin hypersensitivity [14,15]. While they do not replace mechanical tooth brushing, mouthwashes are considered an important adjunct in maintaining oral health, as they allow access to hard-to-reach areas and deliver active substances with specific therapeutic effects [15,16]. Mouthwashes are mixtures of chemical compounds in which the main component is a solvent/carrier (usually water or a water–ethanol system) [17]. They contain ingredients supporting oral hygiene, such as fluoride compounds, potassium compounds, and antimicrobial agents, as well as surfactants, plant extracts, and essential oils that improve taste and aroma and also exhibit antimicrobial and anti-inflammatory properties [14,18,19]. In addition, mouthwashes may include whitening agents such as hydrogen peroxide [20], preservatives, colorants, sweeteners, and humectants that help maintain moisture [21,22,23].

Research indicates that the properties of composite materials used for direct restorations may be altered by exposure to chemical ingredients of mouthwashes. Investigations have been primarily focused on microhardness analyses and the effect of ethanol-containing formulations on surface hardness reduction, yielding inconsistent results [24,25,26], but investigations of mechanical properties at the macroscale, such as diametral tensile strength (DTS) or compressive strength (CS), remain very rare [27,28]. At the same time, it has been reported that the use of mouthwashes may significantly affect the color of composite restorations [29,30].

It is important to note that existing studies have evaluated only a limited number of mouthwashes per investigation, most of which were ethanol-containing formulations, despite the fact that these products now constitute a relatively small segment of the market. This approach has led to an overrepresentation of studies focusing on alcohol-containing mouthwashes, whereas comparative analyses of different types of alcohol-free formulations remain limited. Furthermore, none of the existing studies have comprehensively evaluated the effects of mouthwashes on a broad range of physicochemical properties simultaneously, and the potential impact of mouthwash use on these properties at the macroscopic level has largely been overlooked. Consequently, the current body of evidence does not allow for definitive conclusions as to whether specific groups of mouthwashes may be considered particularly beneficial or detrimental from a clinical perspective in the context of composite restorations. In addition, there is a noticeable lack of studies addressing mouthwashes containing charcoal.

Therefore, the present study aimed to investigate whether long-term exposure to different types of mouthwashes exerts differential effects on the stability of the mechanical and aesthetic properties of a composite material used for direct restorations. The null hypothesis was that exposure to mouthwashes does not result in statistically significant changes in the mechanical and aesthetic properties of the composite restorative material, regardless of formulation.

## 2. Materials and Methods

### 2.1. Materials

A light-curing nanocomposite material, Easy Fill Nano Composite (GDF Gesellschaft für Dentale Forschung und Innovationen GmbH, Köln, Germany), was selected. A material from a brand not considered among the leading ones was selected, it is characterized by good properties as declared by the manufacturer and uses a combination of monomers in a matrix similar to that of many materials [31,32], with comparable declared performance characteristics. This approach was considered economically justified, all the more so since it is difficult to find a material that could be regarded as clearly representative of a specific type of composite or of the entire material group. Its monomer matrix consists of UDMA, 1,4-butanediol dimethacrylate (BDDMA), bis-GMA, and 75 wt% inorganic fillers with particle sizes ranging from 0.04 to 3.0 µm. The specimens were prepared from material of shade A3. All materials originated from a single manufacturing batch.

Twelve mouth rinses were selected for testing. Their chemical compositions, based on manufacturers’ declarations and pH values measured using a pH-meter 410 equipped with a pH electrode ERH-12-6 (Elmetron, Zabrze, Poland), are presented in Table 1. Distilled water was used as the reference medium. Products with different declared functions and chemical compositions were selected. Mouth rinses clearly intended by manufacturers for short-term use, such as Eduril Classic containing 43% (*v*/*v*) ethanol, were excluded despite their expected strong influence on composite properties.

### 2.2. Sample Preparation

The sample preparation procedure was similar for all tests and differed only in specific details. The composite material was packed into a mold placed on a microscope slide, covered with a 50 µm thick polyester foil, and pressed for 30 s through an additional microscope slide to remove excess material. All samples were light-cured using a DY400-4 LED lamp (Denjoy Dental, Changsha, China) emitting light in the 450–470 nm wavelength range with an intensity of 1000–1400 mW/cm^2^.

Specimens for DTS and Vickers microhardness (HV) testing, with a diameter of 6 mm and a height of 3 mm, were irradiated on the upper and lower surfaces [33]. In the case of compression test specimens, with a diameter of 3 mm and a height of 6 mm [34], irradiation was performed on the upper and lower surfaces; after removal from the molds, the specimens were additionally irradiated on the lateral surfaces [35]. Samples for color change evaluation, with a diameter of 12 mm and a thickness of 3 mm, were polymerized using overlapping irradiation zones on the upper and lower surfaces [36,37].

After polymerization, the Teflon plates together with the specimens were wet-ground using P800-grit abrasive paper to remove excess material and to standardize the surfaces, after which the specimens were removed from the molds. For microhardness testing, the surface in contact with the microscope slide was used as the test surface and was left untreated to preserve surface smoothness (ensuring reliable measurements of indentations) and to eliminate the risk of grinding off material layers of varying thickness (ensuring identical initial conditions). Before exposure, all specimens were conditioned in distilled water at 37 °C for 24 h to allow the dark phase of polymerization to occur.

### 2.3. Exposure to Liquids

The specimens were placed in dark 50 mL pharmaceutical glass jars and immersed in 30 mL of the test or control liquid. The sealed jars were mounted in a rotary holder attached to a Hei-Torque 400 overhead stirrer (Heidolph, Schwabach, Germany) and subsequently immersed in a chamber filled with distilled water, maintained at 37 ± 0.5 °C using heating elements and thermostats. The specimens were protected from light. The jars were positioned so that the water level was approximately 1 cm below the screw caps.

The system was set in rotational motion, accelerating from 0 to 150 rpm within 1 min, followed by abrupt stopping and resumption of rotation; these cycles were repeated throughout the entire experiment. This procedure ensured continuous mixing of the solutions within the jars. The stirring intensity was experimentally adjusted to prevent detachment of specimens from the bottom of the jars, which could otherwise result in adhesion between samples and hinder the access of the test liquids to the specimen surfaces used for microhardness and color measurements. In the case of specimens used for compressive strength testing, the selected rotational speed allowed the specimens to move freely; however, due to the nature of the test and the geometry of the specimens, this movement was considered advantageous, as it improved liquid access to the entire specimen surface.

Measurements were performed under baseline conditions and after three 24 h exposure cycles (after 24 h, 48 h, and 72 h) to the test solutions and the reference medium. The exposure duration was selected based on literature data, assuming that 24 h of continuous exposure corresponds approximately to two years of mouthwash use (2 min per day) [38]. For microhardness and color change measurements, the test liquids were replaced every 24 h.

### 2.4. Test Methods

#### 2.4.1. Diametral Tensile Strength Test

The tests were conducted according to a previously described method [38]. After exposure, the specimens were dried with filter paper. For each specimen, the diameter and height were measured at three locations using a digital caliper, and the mean values were used for analysis. Ten specimens were prepared for each medium–exposure time condition, resulting in a total of 520 specimens. Compressive load was applied to the lateral surface at a crosshead speed of 0.5 mm/min using a universal testing machine (Zwick Z020, Zwick GmbH & Co., Ulm, Germany), and the DTS (MPa) values were calculated according to the following equation:

(1) $$DTS=\frac{2F}{\pi dh}\;\;,$$

where *F* is the force at fracture (N), and *d* and *h* are the diameter and thickness (mm), respectively.

#### 2.4.2. Compressive Strength Test

Ten specimens were prepared for each medium–exposure time condition, resulting in a total of 520 specimens. After drying and measuring the specimens as described in Section 2.4.1, the tests were performed at a crosshead speed of 0.5 mm/min using a universal testing machine (Zwick Z020, Zwick GmbH & Co., Ulm, Germany), and the compressive strength (CS, MPa) values were calculated as the fracture force divided by the initial cross-sectional area.

#### 2.4.3. Vickers Hardness Test

For each mouthwash, ten specimens were prepared (130 specimens in total), and four indentations were made in each specimen: one near the center and three approximately 1 mm from the edge at the 12, 4, and 8 o’clock positions. The mean of the four measurements was used as the specimen’s hardness. Measurements were performed using a microhardness tester (Future-Tech FM-700, Future-Tech Corp, Tokyo, Japan) under a 50 g load for 15 s.

#### 2.4.4. Color Stability

Before measurement, the specimens were dried with filter paper. Color changes were evaluated using a spectrophotometer (CM-2600d, Konica Minolta, Tokyo, Japan) according to the CIELAB color system, using a D65 illuminant and a black ceramic background. Measurements were taken at three standardized locations on each specimen, and the mean value was used for analysis. The L* coordinate (lightness, ranging from 0 [black] to 100 [white]), the a* coordinate (red [+a*]–green [−a*]), and the b* coordinate (yellow [+b*]–blue [−b*]) were recorded, and the color difference (ΔE*) was calculated as follows [39]:

(2) $${∆E}^{*}=\sqrt{{{(∆L}^{*})}^{2}+{{(∆a}^{*})}^{2}{{+(∆b}^{*})}^{2}},$$

where ΔL* = *L*_(exposure time)_ − *L*_(baseline)_; Δa* = *a*_(exposure time)_ − *a*_(baseline)_; and Δb* = *b*_(exposure time)_ − *b*_(baseline)_.

#### 2.4.5. Sample Size Calculation and Statistical Analysis

Sample size calculation was performed using the G*Power 3.1 (Heinrich Heine University Düsseldorf, Düsseldorf, Germany). For mechanical properties, the effect size for a one-way ANOVA with four (time effect) and thirteen (medium effect) independent groups was calculated based on pilot data. For CS, DTS, and microhardness, the mean values and standard deviations used ranged from 57 to 65 MPa (SD = 6), 420 to 465 MPa (SD = 23 MPa), and 63 to 80 HV (SD = 7 HV), respectively. A statistical power of 80% and a significance level (α) of 0.05 were assumed [40,41]. The minimum required sample size per group for mechanical testing was as follows: for DTS, 9 (four groups) and 7 (thirteen groups); for CS, 7 and 4; and for microhardness, 5 (for both group configurations). To compensate for potential unexpected results and to increase statistical power, the sample size was increased to n = 10 per group (N = 40 for each medium and N = 130 for each time point). For color measurements, the sample size calculation was based on previously published data [42], and for three groups (time effect), the minimum required sample size was n = 5 (N = 65).

Statistical analyses were performed using PQStat software, version 1.8.0.476 (PQStat Software, Poznan, Poland). The results were analyzed using one-way analysis of variance (ANOVA), with the Brown–Forsythe F* correction applied when the assumption of homogeneity of variances was not met. Homogeneity of variances was assessed using Levene’s test. The normality of data distribution was evaluated using the Shapiro–Wilk test. The level of statistical significance was set at α = 0.05. Tukey’s HSD post hoc test was performed to identify statistically significant differences between groups, with the significance level set at α = 0.05. In addition, correlation analysis of selected properties was conducted. The relationships between the analyzed variables were assessed using Pearson’s correlation coefficient (r) (after testing for normality). Correlation strength was interpreted according to commonly accepted criteria: r < 0.3—weak, 0.3–0.5—moderate, and >0.5—strong correlation [43]. The level of statistical significance was set at α = 0.05.

## 3. Results

### 3.1. Mechanical Properties

**Figure 1:**
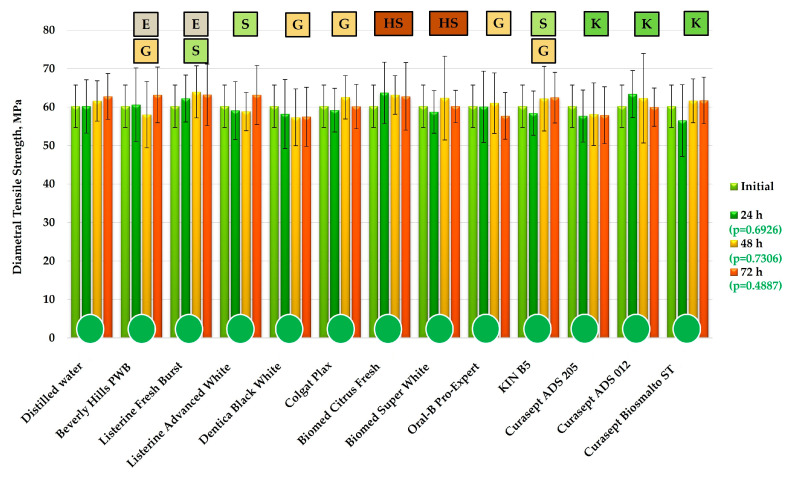
The DTS changes (mean values and standard deviations) after exposure to mouthwashes and distilled water. The *p*-values refer to the effect of the mouthwashes at a given exposure time. Letters indicate components in the mouthwash formulations: E—ethanol, G—glycerol, S—sorbitol, K—xylitol, HS—hydrogenated starch hydrolysate. Green circles indicate the absence of statistically significant changes over time during exposure to the different liquids (α = 0.05).

The DTS results are presented in Figure 1. No statistically significant differences in the mean DTS values were observed after exposure to the mouthwashes for 24, 48, or 72 h. Furthermore, DTS values remained stable over time, with no significant effect of increasing exposure time to any of the tested liquids.

**Figure 2:**
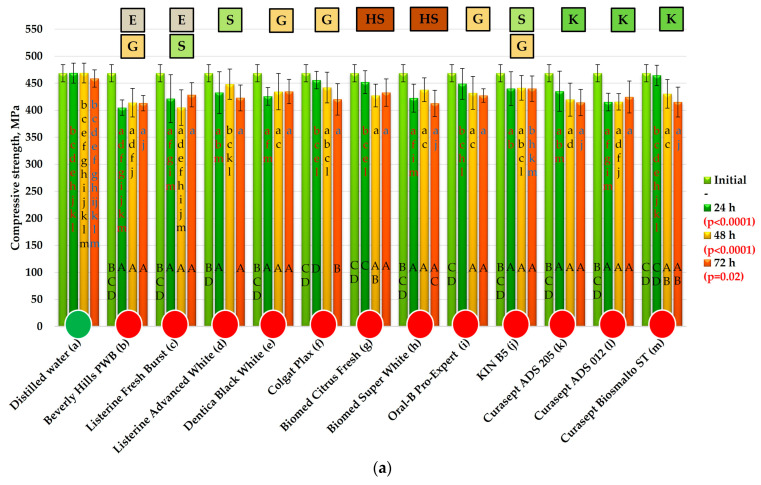

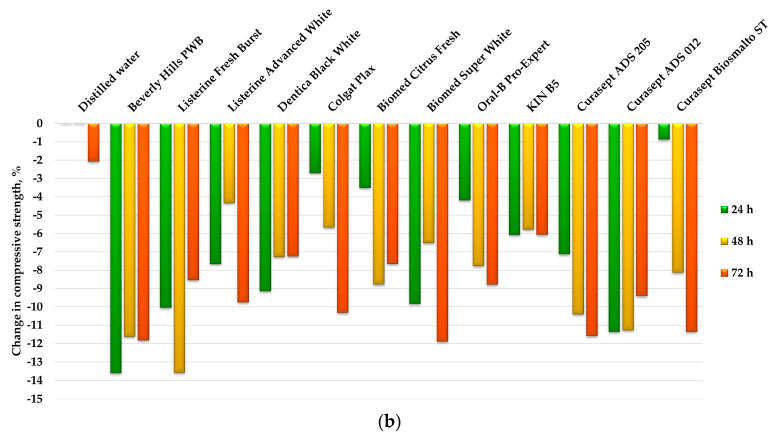
The compressive strength changes (mean values and standard deviations) after exposure to mouthwashes and distilled water (**a**), and the percentage changes in mean values relative to the initial value (**b**). The *p*-values refer to the effect of the mouthwashes at a given exposure time. Lowercase letters (a–m) on the bars indicate that the mean value was statistically significantly different (Tukey’s HSD test, α = 0.05) compared with the value obtained for the mouthwash marked with the same letter on the axis at a given exposure time. Circles indicate the absence (green) or presence (red) of statistically significant changes over time (α = 0.05). Uppercase letters (A–D) on the bars refer to consecutive exposure times for a given mouthwash; the presence of the same letter indicates a statistically significant difference. Letters indicate components in the mouthwash formulations: E—ethanol, G—glycerol, S—sorbitol, K—xylitol, HS—hydrogenated starch hydrolysate.

The results of the compressive strength (CS) measurements together with the statistical analysis are presented in Figure 2. The mean compressive strength of the composite material before exposure to liquids was 468 ± 16 MPa.

After 24 h of exposure, the highest mean compressive strength was recorded for the control medium (distilled water, 469 MPa), whereas the lowest value was observed after exposure to Beverly Hills PWB mouthwash (405 MPa). In most cases, the mean CS values obtained after exposure to mouthwashes were statistically significantly different compared with those measured after exposure to distilled water. No statistically significant differences were registered only for four mouthwashes: Colgate Plax, Biomed Citrus Fresh, Oral-B Pro-Expert, and Curasept Biosmalto Sensitive Teeth. Considerable variability in the results was also evident, as for three mouthwashes (including both ethanol-containing formulations) the reduction in CS exceeded 10%, whereas for four mouthwashes (those not significantly different from the control) the decrease did not exceed 4%. Moreover, the mean CS after exposure to Beverly Hills PWB was statistically significantly lower than that obtained after exposure to seven other mouthwashes.

After 48 h of exposure, the highest mean CS was again recorded for distilled water (469 MPa), while the lowest value was recorded after exposure to Listerine Fresh Burst (405 MPa). For the ethanol-containing mouthwashes, Beverly Hills PWB and Listerine Fresh Burst, the greatest reductions in mean CS were observed (12% and 14%, respectively). In the case of Beverly Hills PWB, the CS value was statistically significantly lower than that obtained after storage in three other mouthwashes, whereas for Listerine Fresh Burst it was significantly lower than after exposure to seven other mouthwashes. For Curasept ADS 205 and Curasept ADS 012, the reduction in CS relative to distilled water exceeded 10%, while for the remaining mouthwashes, the mean CS values were lower than those of the reference medium by 4% to 9%.

After 72 h of exposure, the highest compressive strength was observed for distilled water (459 MPa), whereas the lowest was observed after exposure to Beverly Hills PWB (412 MPa). Differences in the effects of individual mouthwashes on CS were less pronounced at this time point. Statistically significant differences in CS were observed only between KIN B5 and four mouthwashes (Beverly Hills PWB, Biomed Super White, Curasept ADS 205, and Curasept Biosmalto Sensitive Teeth). Compared with the control group, the reduction in mean CS ranged from 6% (KIN B5) to 12%.

Exposure to water did not cause significant changes in CS. For eight mouthwashes (Beverly Hills PWB, Listerine Fresh Burst, Listerine Advanced White, Dentica Black White, Biomed Super White, KIN B5, Curasept ADS 205, Curasept ADS 012), a statistically significant decrease in CS was observed after 24 h compared with baseline; however, prolonging the exposure did not result in a further significant reduction in CS (except Biomed Super White).

For the remaining four mouthwashes, CS decreased significantly only after 48 h, and the process of CS reduction intensified with increasing exposure time for three of them (Colgate Plax, Biomed Citrus Fresh, Curasept Biosmalto Sensitive Teeth).

**Figure 3:**
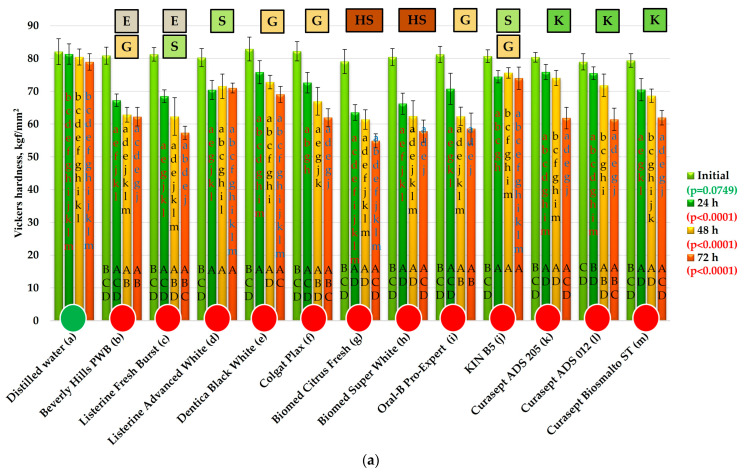

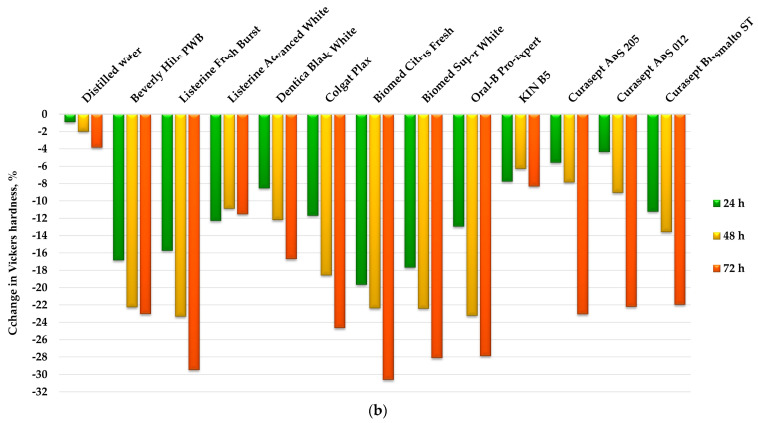
The Vickers hardness changes (mean values and standard deviations) after exposure to mouthwashes and distilled water (**a**), and the percentage changes in mean values relative to the initial value (**b**). The *p*-values refer to the effect of the mouthwashes at a given exposure time. Lowercase letters (a–m) on the bars indicate that the mean value was statistically significantly different (Tukey’s HSD test, α = 0.05) compared with the value obtained for the mouthwash marked with the same letter on the axis at a given exposure time. Circles indicate the absence (green) or presence (red) of statistically significant changes over time (α = 0.05). Uppercase letters (A–D) on the bars refer to consecutive exposure times for a given mouthwash; the presence of the same letter indicates a statistically significant difference. Letters indicate components in the mouthwash formulations: E—ethanol, G—glycerol, S—sorbitol, K—xylitol, HS—hydrogenated starch hydrolysate.

The results of the HV measurements and statistical analyses are presented in Figure 3. Before exposure, the mean HV values for the control and experimental groups ranged from 79 to 82.9 kgf/mm^2^, with no statistically significant differences observed (*p* = 0.0749), confirming appropriate sample preparation.

After 24 h of exposure, all mouthwashes caused a statistically significant decrease in hardness compared with the reference solution. The reduction ranged from 4% (Curasept ADS 012) to 20% (Biomed Citrus Fresh). For KIN B5 and the chlorhexidine-containing mouthwashes (Curasept ADS 205 and Curasept ADS 012), the decrease in mean HV did not exceed 8%, while for Dentica Black White it reached 8.5%. In the remaining mouthwashes, the reduction exceeded 10%, including 16% for Beverly Hills Perfect WB and 17% for Listerine Fresh Burst (ethanol-containing mouthwashes), as well as 18% for Biomed Super White and 20% for Biomed Citrus Fresh (mouthwashes based on 98% natural ingredients). No statistically significant correlation was observed between solution pH and percentage hardness change (*p* = 0.845, r = −0.06). The very low correlation coefficient indicates a negligible relationship, implying that pH did not have a measurable practical effect on hardness changes in the tested system.

After 48 h, the mean HV values ranged from 61 kgf/mm^2^ (Biomed Citrus Fresh) to 76 kgf/mm^2^ (KIN B5), corresponding to hardness reductions between 6% and 23%. For KIN B5, Curasept ADS 205, and Curasept ADS 012, the reduction in mean HV did not exceed 10%. For the remaining five mouthwashes (Beverly Hills PWB, Listerine Fresh Burst, Biomed Citrus Fresh, Biomed Super White, and Oral-B Pro-Expert), the results were comparable and ranged from 61.4 to 62.4 kgf/mm^2^, corresponding to reductions of 22–23%. No statistically significant correlation was found between pH values and hardness reduction (*p* = 0.3701, r = 0.27). Although the correlation coefficient suggests a weak positive trend, the relationship is not statistically significant and therefore does not indicate a meaningful practical influence of pH on hardness reduction.

After 72 h, all mouthwashes showed statistically significant differences compared with the control sample (78.9 kgf/mm^2^). The highest mean hardness was recorded for KIN B5 (74 kgf/mm^2^), representing an 8% decrease relative to baseline. The value for KIN B5 differed significantly only from that obtained for Listerine Advanced White (71 kgf/mm^2^), for which the reduction exceeded 10%. No significant differences were found between Listerine Advanced White and Dentica Black White (69.1 kgf/mm^2^). For the remaining mouthwashes, the reduction in mean HV exceeded 20%, reaching 28% for Biomed Super White and Oral-B Pro-Expert, 30% for Listerine Fresh Burst, and 31% for Biomed Citrus Fresh. No statistically significant correlation was observed between solution pH and the percentage change in hardness (*p* = 0.2548, r = 0.34). The correlation coefficient suggests a weak positive association; however, the relationship is not statistically significant and therefore indicates no meaningful practical effect of pH on hardness change.

As a result of sample exposure to DW, no statistically significant differences in mean HV values were observed, and the mean hardness decreased by 4% (3.2 kgf/mm^2^) over the course of the experiment. All tested mouthwashes caused a statistically significant reduction in mean hardness values. For eleven mouthwashes (Beverly Hills PWB, Listerine Fresh Burst, Listerine Advanced White, Dentica Black White, Colgate Plax, Biomed Citrus Fresh, Biomed Super White, Oral-B Pro-Expert, KIN B5, and Curasept Biosmalto Sensitive Teeth), the greatest decrease in mean hardness was observed within the first 24 h of the experiment. This initial reduction was generally comparable to, or in some cases clearly greater than (Listerine Advanced White, Dentica Black White), the cumulative decrease resulting from extending the exposure by a further 48 h (from 24 h to 72 h).

Eleven out of twelve mouthwashes caused statistically significant changes in hardness after 24 h of exposure, with Curasept ADS 012 being the only exception. For Curasept ADS 012 and Curasept ADS 205, the greatest decrease in hardness was recorded during the final 24 h of exposure. Most mouthwashes induced a progressive and statistically significant reduction in hardness during the first two days or throughout the entire experimental period. Only Listerine Advanced White and KIN B5 did not cause a statistically significant decrease in mean HV values after the first 24 h of exposure.

### 3.2. Color Changes

**Figure 4:**
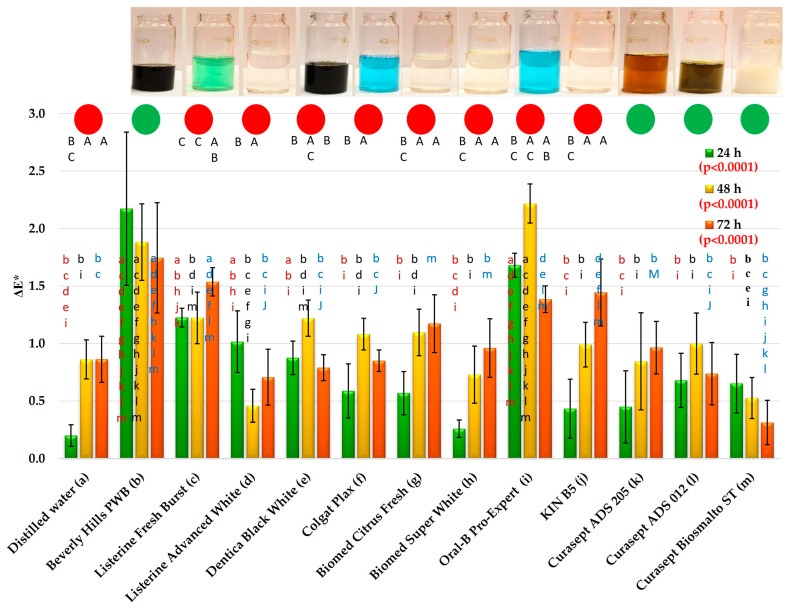
The ΔE* changes (mean values and standard deviations). The *p*-values refer to the effect of the mouthwashes at a given exposure time. Lowercase letters (a–m) on the bars indicate that the mean value was statistically significantly different (Tukey’s HSD test, α = 0.05) compared with the value obtained for the mouthwash marked with the same letter on the axis at a given exposure time. Circles indicate the absence (green) or presence (red) of statistically significant changes over time (α = 0.05). Uppercase letters (A–C) above the bars refer to consecutive exposure times for a given mouthwash; the presence of the same letter indicates a statistically significant difference.

After 24 h of exposure to the control medium and the mouthwashes, statistically significant differences in the mean ΔE values were observed (Figure 4). Following exposure to distilled water (DW), the mean ΔE was 0.2, whereas for the mouthwashes it ranged from 0.26 (Biomed Super White) to 2.17 (Beverly Hills PWB). Post hoc analysis revealed that for seven mouthwashes (Colgate Plax, Biomed Super White, Biomed Citrus Fresh, KIN B5, Curasept ADS 205, Curasept ADS 012, Curasept Biosmalto ST), the obtained results did not differ statistically significantly from those for DW. In contrast, the use of Beverly Hills PWB resulted in statistically significant differences compared with all other solutions except Oral-B Pro-Expert.

For Oral-B Pro-Expert, the mean ΔE value of 1.68 was also statistically significantly higher than that observed for most mouthwashes (no significant differences were found only in comparison with Listerine Fresh Burst and Beverly Hills PWB Burst). Mean ΔE values ≥ 1 were additionally recorded for Listerine Fresh Burst and Listerine Advanced White; no statistically significant differences were found between them. Moreover, these values did not differ significantly from the mean ΔE of 0.88 obtained for Dentica Black White.

For the Colgate Plax group, Biomed Citrus Fresh, Curasept ADS 012, Curasept ADS 205, and Curasept Biosmalto Sensitive Teeth, mean ΔE values ranged from 0.57 to 0.68, with no statistically significant differences among them.

After 48 h of exposure to DW, the mean ΔE was 0.86, while for the mouthwashes it ranged from 0.46 (Listerine Advanced White) to 2.22 (Oral-B Pro-Expert). Post hoc analysis demonstrated that only two mouthwashes produced results statistically significantly different from the control liquid (Oral-B Pro-Expert and Beverly Hills PWB). The mean ΔE value following treatment with Oral-B Pro-Expert did not differ significantly only from that obtained with Beverly Hills PWB; however, the results for these two mouthwashes were statistically higher than those for all remaining solutions.

Additionally, mean ΔE values ≥ 1 were observed after the use of six other mouthwashes (Listerine Fresh Burst, Dentica Black White, Colgate Plax, Oral-B Pro-Expert, Biomed Citrus Fresh, Curasept ADS 012), while for KIN B5 the mean value was 0.99. Within this group, no statistically significant differences were noted. For the remaining four mouthwashes (Listerine Advanced White, Biomed Super White, Curasept ADS 205, Curasept Biosmalto Sensitive Teeth), mean ΔE values ranged from 0.46 to 0.82 and did not differ significantly from one another.

After 72 h of exposure to DW, the mean ΔE was 0.86, whereas for the mouthwashes it ranged from 0.31 (Curasept Biosmalto Sensitive Teeth) to 1.75 (Beverly Hills PWB). Statistical analyses demonstrated that for two mouthwashes (Listerine Fresh Burst and Beverly Hills PWB), the results differed significantly from those obtained for water. Mean ΔE values > 1 were recorded after exposure to five mouthwashes (Beverly Hills PWB, Listerine Fresh Burst, Biomed Citrus Fresh, Oral-B Pro-Expert, KIN B5). Slightly lower values (*p* > 0.05) were noted for Biomed Super White (0.96) and Curasept ADS 205 (0.96). For Listerine Advanced White, Dentica Black White, Colgate Plax, Biomed Super White, Curasept ADS 205, and Curasept ADS 012, mean color change values were above 0.5 and below 1.

Exposure time had a statistically significant effect on ΔE values for eight mouthwashes and for water; however, no statistically significant changes over time were observed for all Curasept mouthwashes and for Beverly Hills PWB. For DW, statistically significant differences were found between 24 and 48 h of the experiment, after which the values stabilized. Similar results were observed for Biomed Citrus Fresh, Biomed Super White, and KIN B5. Following the use of Listerine Fresh Burst, elevated ΔE values persisted for 48 h and increased further at 72 h. For Oral-B Pro-Expert, an increase in mean ΔE was observed between 24 and 48 h, followed by a decrease between 48 and 72 h. In the case of Beverly Hills PWB, Listerine Advanced White, and Curasept Biosmalto Sensitive Teeth, the highest mean ΔE values were recorded after 24 h of exposure, with a subsequent statistically significant reduction observed for two of these mouthwashes.

**Figure 5:**
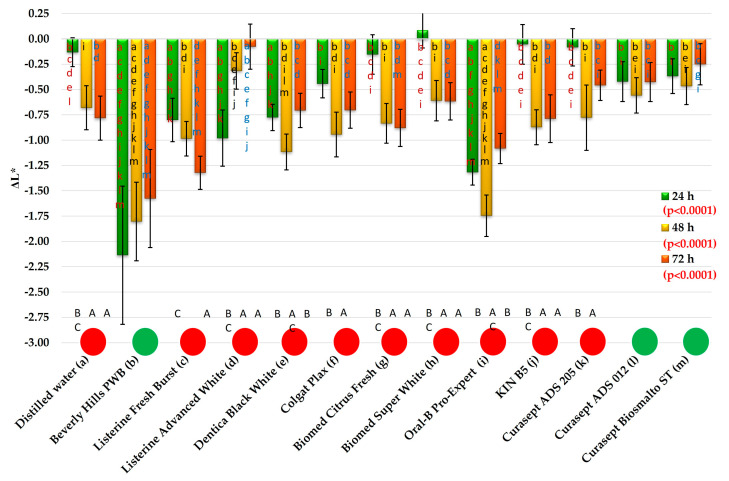
The ΔL* changes (mean values and standard deviations). The *p*-values refer to the effect of the mouthwashes at a given exposure time. Lowercase letters (a–m) on the bars indicate that the mean value was statistically significantly different (Tukey’s HSD test, α = 0.05) compared with the value obtained for the mouthwash marked with the same letter on the axis at a given exposure time. Circles indicate the absence (green) or presence (red) of statistically significant changes over time (α = 0.05). Uppercase letters (A–C) below the bars refer to consecutive exposure times for a given mouthwash; the presence of the same letter indicates a statistically significant difference.

The ΔL* changes were presented in Figure 5. After 24 h of exposure to distilled water (DW), the mean ΔL* value was −0.13, whereas for the mouthwashes it ranged from −0.05 (KIN B5) to −2.14 (Beverly Hills PWB). For five mouthwashes (Beverly Hills PWB, Listerine Fresh Burst, Listerine Advanced White, and Oral-B Pro-Expert), the obtained results were statistically significantly different from those recorded for water. Mean ΔL* values lower than −1 (absolute value > 1) were observed for Beverly Hills PWB and Oral-B Pro-Expert, while for Listerine Advanced White the mean value was −0.98. Among the remaining mouthwashes, mean ΔL* values lower than −0.5 (absolute value > 0.5) were recorded for Listerine Fresh Burst (−0.8) and Dentica Black White (−0.78). For the other mouthwashes, the absolute ΔL* value was below 0.5.

The mean ΔL* value obtained after the application of Beverly Hills PWB was statistically significantly higher than those recorded after exposure to all other tested liquids. No statistically significant difference in ΔL* was found between Listerine Advanced White and Oral-B Pro-Expert. The mean ΔL* values obtained after exposure to Listerine Fresh Burst, Listerine Advanced White, and Dentica Black White (absolute values close to 1) did not differ significantly from one another. The ΔL* value obtained for KIN B5 (the lowest absolute value) did not differ significantly from those recorded for six other mouthwashes and DW.

After 48 h of exposure to DW, the mean ΔL* value was −0.68, whereas for the mouthwashes it ranged from −0.32 (Listerine Advanced White) to −1.8 (Beverly Hills PWB). For only three mouthwashes (Beverly Hills PWB, Dentica Black White, and Oral-B Pro-Expert), the mean absolute ΔL* values exceeded 1, while for Listerine Fresh Burst the mean value was 0.99. However, post hoc analysis demonstrated that only the results obtained for Beverly Hills PWB and Oral-B Pro-Expert were significantly higher than those recorded for water. The mean ΔL* values obtained after exposure to Beverly Hills PWB and Oral-B Pro-Expert did not differ significantly from each other, and their absolute values were higher than those observed for all other mouthwashes.

After 72 h of exposure, the mean ΔL* for DW was −0.78, whereas for the mouthwashes it ranged from −0.08 (Listerine Advanced White) to −1.32 (Listerine Fresh Burst).

Exposure to Beverly Hills PWB resulted in a significant increase in ΔL* compared with DW, whereas Listerine Advanced White produced ΔL* values significantly lower than those observed after conditioning in water (indicating a lighter material). For Beverly Hills PWB, Listerine Fresh Burst, and Oral-B Pro-Expert, the absolute ΔL* values exceeded 1 (negative direction) and did not differ significantly from one another.

Exposure time did not significantly affect ΔL* only in the case of Beverly Hills PWB, Curasept ADS 012, and Curasept Biosmalto Sensitive Teeth; however, it should be noted that discoloration caused by the first of these was significantly greater. Post hoc statistical analysis demonstrated that in the case of distilled water, despite the low initial mean ΔL* value, a significant decrease in lightness was observed after 48 h. A similar pattern of ΔL* changes was observed for Listerine Advanced White, Biomed Citrus Fresh, Biomed Super White, and KIN B5.

The use of Listerine Fresh Burst resulted in relatively high absolute ΔL* values after 24 h of the experiment, followed by their gradual increase. Following exposure to Dentica Black White and Oral-B Pro-Expert, high mean ΔL* values were obtained after the first 24 h; their absolute values increased significantly after 48 h and subsequently decreased after 72 h to levels comparable to those observed at 24 h. Exposure to Beverly Hills PWB and Listerine Advanced White produced high absolute ΔL* values after 24 h (increase in absolute value, negative ΔL* values), followed by a gradual and statistically significant decrease; however, in the case of the mouthwash containing activated charcoal, the changes in lightness were markedly greater.

**Figure 6:**
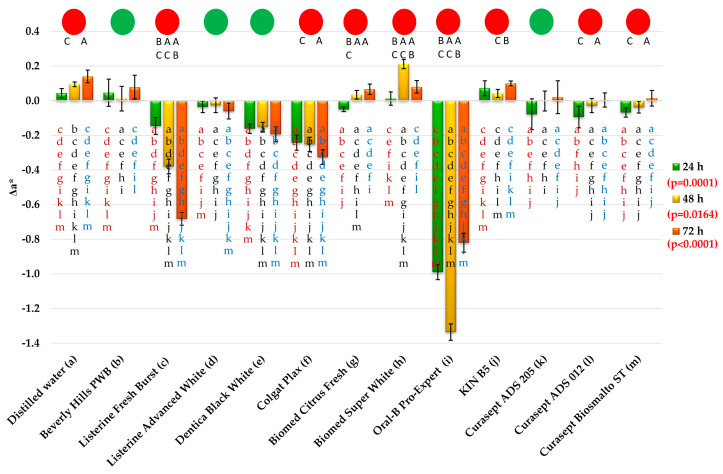
The Δa* changes (mean values and standard deviations. The *p*-values refer to the effect of the mouthwashes at a given exposure time. Lowercase letters (a–m) on the bars indicate that the mean value was statistically significantly different (Tukey’s HSD test, α = 0.05) compared with the value obtained for the mouthwash marked with the same letter on the axis at a given exposure time. Circles indicate the absence (green) or presence (red) of statistically significant changes over time (α = 0.05). Uppercase letters (A–C) above the bars refer to consecutive exposure times for a given mouthwash; the presence of the same letter indicates a statistically significant difference.

Changes in Δa* values are presented in Figure 6. After exposure to distilled water (DW), the mean Δa* value was 0.04, whereas for the mouthwashes the values ranged from 0.01 (Biomed Super White) to −0.99 (Oral-B Pro-Expert).

For five mouthwashes (Beverly Hills PWB, Listerine Advanced White, Biomed Super White, and KIN B5), the results did not differ statistically significantly from those obtained for water. The mean Δa* values after storage in Oral-B Pro-Expert were significantly higher than those recorded for all other tested liquids. For the remaining mouthwashes, Δa* values did not exceed 0.24.

After 48 h of exposure to DW, Δa* amounted to 0.09, whereas for the mouthwashes the mean absolute values ranged from 0 (Curasept ADS 205) to 1.34 (negative direction) for Oral-B Pro-Expert. For the majority of mouthwashes, the obtained results differed statistically significantly compared with those for water (with the exception of KIN B5). The mean value recorded after exposure to Oral-B Pro-Expert differed significantly from all other groups. The mean Δa* value after exposure to Biomed Super White was 0.21, whereas after the application of Colgate Plax and Listerine Fresh Burst it was −0.25 and −0.38, respectively; these results differed not only from each other but also from all other mouthwashes. For eight of the tested liquids, the mean absolute Δa* values were below 0.15 (with varying directions of change).

After 72 h, Δa* for DW was 0.14, whereas for the mouthwashes it ranged from 0.01 (Curasept ADS 012) to −0.82 (Oral-B Pro-Expert). Changes in Δa* for Beverly Hills Perfect WB, Biomed Citrus Fresh, and KIN B5 did not differ from those obtained for DW. The mean Δa* values were −0.82 for Oral-B Pro-Expert, −0.68 for Listerine Fresh Burst, −0.32 for Colgate Plax, and −0.19 for Dentica Black White 205; these results differed from one another and from all other tested liquids. For the remaining mouthwashes, the mean absolute Δa* values were ≤0.1 (with varying directions of change).

Exposure time significantly influenced Δa* changes for nine out of the thirteen tested liquids. Despite the presence of statistically significant differences for the vast majority of mouthwashes, the recorded changes were generally small. Changes observed for Listerine Fresh Burst (from −0.15 to −0.68) and Oral-B Pro-Expert may be considered practically relevant, not only statistically significant.

**Figure 7:**
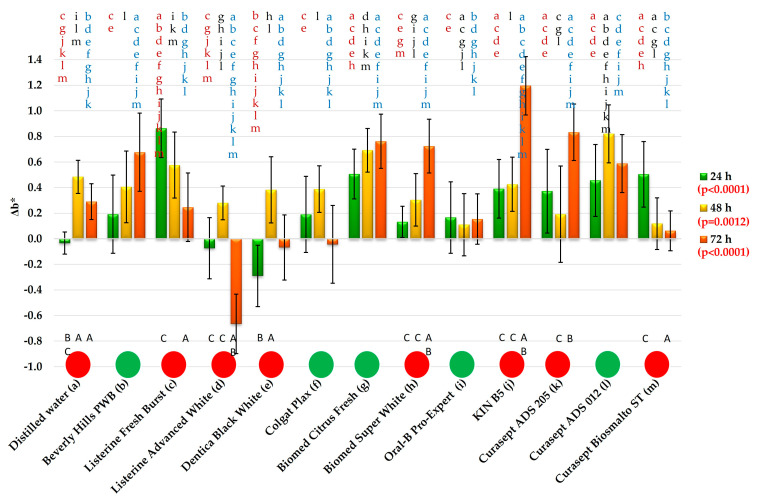
The Δb* changes (mean values and standard deviations. The *p*-values refer to the effect of the mouthwashes at a given exposure time. Lowercase letters (a–m) on the bars indicate that the mean value was statistically significantly different (Tukey’s HSD test, α = 0.05) compared with the value obtained for the mouthwash marked with the same letter on the axis at a given exposure time. Circles indicate the absence (green) or presence (red) of statistically significant changes over time (α = 0.05). Uppercase letters (A–C) below the bars refer to consecutive exposure times for a given mouthwash; the presence of the same letter indicates a statistically significant difference.

After 24 h of exposure to distilled water (DW), the mean Δb* was −0.03, whereas for the mouthwashes the values ranged from −0.07 (Listerine Advanced White) to 0.86 (Listerine Fresh Burst). Post hoc analysis demonstrated (Figure 7) that for six mouthwashes the results did not differ significantly from DW; only Listerine Fresh Burst showed a statistically significant difference compared with water (Listerine Advanced White, Dentica Black White, Colgate Plax, Biomed Super White, Oral-B Pro-Expert, Beverly Hills Perfect White). The highest mean value (Listerine Fresh Burst) differed significantly from all other rinses, whose mean values were within a narrow range from 0.07 (Listerine Advanced White) to 0.51 (Biomed Citrus Fresh). For five mouthwashes (Beverly Hills PWB, Listerine Advanced White, Biomed Super White, Colgate Plax, Oral-B Pro-Expert), the absolute mean Δb* values were ≤0.2, with variable directions of change.

After 48 h, the mean Δb* following exposure to DW was 0.48, while for the mouthwashes the values ranged from 0.11 (Oral-B Pro-Expert) to 0.82 (Curasept ADS 012). Statistically significant differences compared with DW were observed for only three mouthwashes: Oral-B Pro-Expert, Curasept Biosmalto ST, and Curasept ADS 012; in the first two cases the values were lower than for water, whereas in the latter they were higher.

After 72 h of storage in distilled water, the mean Δb* was 0.29, whereas for the mouthwashes the absolute mean values ranged from 0.04 (negative direction; Colgate Plax) to 1.2 (KIN B5). For four mouthwashes (Listerine Fresh Burst, Oral-B Pro-Expert, Curasept Biosmalto ST, and Curasept ADS 012), no significant differences were found compared with DW.

For Listerine Advanced White, the mean Δb* of −0.67 was the only distinctly negative value, whereas for three mouthwashes (Dentica Black White, Colgate Plax, Curasept Biosmalto ST) the absolute mean Δb* values did not exceed 0.01. Significant differences compared with water (positive direction of change) were observed for Beverly Hills PWB, Biomed Citrus Fresh, Biomed Super White, KIN B5, and Curasept ADS 205. The mean Δb* exceeded 1 only for KIN B5, while for five other rinses the absolute mean values ranged between 0.5 and 1.

Exposure time had a statistically significant effect on Δb* for distilled water and seven mouthwashes. In the case of water, the changes occurred in a positive direction. Time-dependent changes were also observed for Listerine Fresh Burst, Listerine Advanced White, and Curasept Biosmalto ST. For Biomed Super White, KIN B5, and Curasept ADS 205, the changes were positive, with particularly pronounced effects on the final day of the experiment.

## 4. Discussion

Mouthwashes are widely used in daily oral hygiene and have previously been analyzed in terms of their interactions with dental composites. Previous studies on the impact of mouthwashes on dental composites were conducted using from one to several composite materials and at most a few mouthwashes within a single study [25,26,44,45,46,47,48]. Although the results differed in detail for various composites, they generally indicated similar trends [25,44,46]. The occurrence of such a pattern is understandable when considering the frequent similarities in the compositions of commercially available composite materials, both in terms of resin matrices and the types of fillers and additional components used. For this reason, we selected a single composite material with a traditional composition and a broad spectrum of clinical applications. It should be noted, however, that this choice does not imply that the material can be considered fully representative of all composites, due in part to the relatively limited number of independent studies describing its properties. Simultaneously a substantially greater number of mouthwashes (12) were chosen than in previous studies, including products belonging to different groups in terms of chemical composition and/or intended action (e.g., more than one ethanol-containing mouthwash, more than one containing chlorhexidine, with charcoal, fluorides, etc.).

The study used specimens made of a material in shade A3, which is the most commonly encountered tooth shade in clinical practice. Elamin et al. [49] reported that teeth of shade A3 were characteristic of 36.1% of the examined subjects, whereas A2 accounted for 27.3% and A1 for 11.5%. Distilled water was used as the reference medium, as a preliminary test demonstrated that the selected properties changed to the same extent as in artificial saliva, making distilled water a more convenient medium. The exposure time was determined based on literature data, assuming that 24 h of exposure approximately corresponds to two years of daily mouthwash use (2 min per day) [38,50].

Attention was also given to the method of specimen surface preparation due to the potential influence of surface roughness on interactions with the tested liquids [51]. Typical Ra values obtained after finishing and polishing procedures performed in a dental office depend on the polishing system and range from 0.1 µm to 0.6 µm, most commonly between 0.25 µm and 0.4 µm [52,53,54,55,56]. Therefore, based on a preliminary experiment involving abrasive papers of various grit sizes, P800 grit was selected, as it enabled the achievement of Ra values of approximately 0.35 µm. Samples for microhardness testing were not subjected to additional surface treatment, which allowed the preservation of the primary surface quality determined by contact with microscopic glass. This ensured accurate measurement of the indentation diagonals and maintained fully comparable characteristics of the surface layers.

Changes in microhardness are the most frequently analyzed mechanical property of composite materials in the context of mouthwash application. In the present study, a load of 50 g was applied, resulting in indentations with diagonal lengths of approximately 30–40 µm and a depth of approximately 5–6 µm. Thus, the analyzed micro-areas were sufficiently large to remain representative with respect to the filler particle size, while the indentation depth enabled assessment of the surface properties. The baseline hardness values were consistent with those declared by the manufacturer. For the vast majority of mouthwashes, the greatest changes in hardness were registered after the first 24 h of exposure. An exception was noted for Curasept mouthwashes containing chlorhexidine, for which the greatest reduction in hardness occurred between 48 h and 72 h of the experiment. Moreover, it should be emphasized that these products are primarily recommended by the manufacturer for short-term use (although prolonged use is not excluded); therefore, their performance in contact with the investigated composite was highly satisfactory. After the first 24 h, the most pronounced decrease in composite microhardness was observed for two ethanol and sugar alcohol-containing mouthwashes (Listerine Fresh Burst, Beverly Hills BW), as well as for two Biomed mouthwashes containing hydrogenated starch hydrolysate as the second listed ingredient. Hydrogenated starch hydrolysate is a mixture of various sugar alcohols, such as glycol, glycerol, xylitol, and sorbitol, commonly used as sweeteners with low cariogenic potential [57,58]. However, the role of these compounds in mouthwashes is more complex: glycerol, sorbitol, and xylitol function as sweetening agents influencing taste perception; additionally, xylitol exhibits cariostatic properties, whereas glycerol and sorbitol act as humectants (moisturizing agents) [21,58,59,60].

At a later stage of the experiment (after 48 h), a particularly marked decrease in hardness was observed for mouthwashes containing glycerol as the second listed ingredient, and a somewhat less pronounced decrease for a third glycerol-containing mouthwash (Dentica Black White). This may indicate a significant role of glycerol in reducing composite hardness. After 72 h, a clearly intensified decrease in hardness was also observed following exposure to Curasept products (with xylitol listed as the second ingredient). The least pronounced changes in hardness were recorded for mouthwashes containing sorbitol as the second listed ingredient (Listerine Advanced White, KIN B5).

These findings suggest that the presence of sugar alcohols in mouthwashes may contribute to a reduction in composite hardness to a degree comparable to that induced by ethanol (an alkanol). This is a relevant observation, as numerous previous studies have suggested that ethanol is a component particularly responsible for hardness degradation of composite materials [38,46,61,62]. Although the present study confirms that ethanol induces significant changes in hardness, it simultaneously demonstrates that a wide range of ethanol-free mouthwashes may produce an analogous or very similar effect. Overall, these results are also consistent with observations reported in other studies [24,25], in which, however, the potential role of sugar alcohols in this process was not specifically addressed.

Due to the lack of detailed information from manufacturers regarding the concentrations of individual ingredients in the tested mouthwashes, the observed hardness reduction cannot be directly correlated with the concentration of sugar alcohols. Therefore, any potential relationship between the structure of sugar alcohols and the observed changes in hardness should be interpreted with caution. It may be tentatively hypothesized that shorter-chain sugar alcohols could be associated with more pronounced changes in hardness during the initial phase of exposure, whereas relatively minor hardness changes might occur in the presence of sorbitol (in the absence of other polyols), which has one of the longest carbon chains among sugar alcohols commonly used in mouthwashes. However, this possible relationship remains speculative and has not been experimentally verified. This issue has not been previously investigated and therefore requires systematic and detailed studies to determine whether the rate of hardness degradation of dental composites—or their resin matrices alone—can indeed be related to the concentration and chain length of sugar alcohols. Some previous studies suggested that a relationship exists between pH value and hardness reduction [48,63,64] due to the potential effect of acidic media on the polymer matrices through catalysis of ester groups derived from dimethacrylate monomers. Hydrolysis of ester bonds could lead to the formation of ethanol and carboxylic acid molecules, thereby accelerating matrix degradation [14,38,65]. However, in the present study, based on a substantially larger number of mouthwashes than previously investigated, only weak and statistically insignificant positive trends were observed, and a clear correlation was not confirmed. This is consistent with studies indicating the absence of an unequivocal relationship in this regard [66,67] and suggests that pH alone is not the sole factor determining hardness degradation under the studied conditions. Also, the suggested prevention in hardness reduction by sodium fluoride [26] was not confirmed, because this ingredient was present in the formulations of mouthwashes that exhibited both relatively minor and the most pronounced changes in hardness.

It therefore appears that the primary mechanism responsible for the decrease in composite hardness following exposure to mouthwashes was plasticization of the polymer matrix caused by the penetration of, for example, alcohol molecules between polymer chains. This may result in physical separation of the chains and weakening of intermolecular interactions (bonds) between them [68]. Assuming this mechanism to be dominant could explain the hypothetical relationship between the rate of hardness change and the molecular size of sugar alcohols. Nevertheless, it should be taken into account that other components of individual mouthwashes may also have contributed to the observed hardness changes. Formulating far-reaching conclusions in this regard is difficult due to the complexity and variability of their chemical compositions.

Ultimately, the reduction in microhardness observed following the use of mouthwashes—except for those containing sorbitol listed as the second ingredient—exceeded 15% and ranged from 20% to 30%. These values should be considered significant, particularly in light of the reported correlations between composite hardness and its tribological wear [69,70]. The available data indicate a potential shortening of the functional lifespan of restorations, especially for composites with relatively low baseline hardness. From a clinical perspective, a substantial loss of hardness over a short period (regardless of any potential subsequent stabilization) appears particularly unfavorable, since restorations in service are subjected to alternating exposure to liquids (including mouthwashes) and mechanical wear. Consequently, rapid softening of the surface layer may accelerate its wear and promote repeated softening of the newly exposed surface.

Compressive strength is a clinically important property of dental composites, as it reflects the material’s ability to resist the predominantly compressive forces generated during mastication [71]. Diametral tensile strength (DTS) is widely recognized in the dental materials literature as a mechanical property that relates not only to tensile stresses occurring during mastication and parafunctional activities, but also to stresses resulting from material shrinkage [72,73]. Tensile stresses are particularly important because occlusal forces generate tension not only within the restorative material, but especially at the tooth–restoration interface [74,75]. The ability of a composite to withstand such stresses without fracturing is therefore critical to the long-term durability of a restoration, especially considering that damage formation in this zone is a primary reason for the replacement of dental fillings [76,77]. Consequently, tensile strength is often regarded as more clinically relevant than compressive strength, particularly in areas near the tooth–composite interface [73,78]. In this context, any potential reduction in DTS of a composite should be considered particularly disadvantageous. Analysis of the composite’s mechanical properties at the macro scale revealed no significant changes in diametral tensile strength (DTS), which should be considered desirable and beneficial. A statistically significant reduction in compressive strength (CS) was observed in all cases. For eight mouthwashes, significant changes occurred within the first 24 h of exposure, with no further deterioration detected thereafter. The greatest reductions (>10%) were recorded for mouthwashes containing ethyl alcohol. In contrast, for the remaining four mouthwashes, a significant decrease in CS was observed only after 48 h of exposure. Assuming a compressive strength of approximately 230 MPa as a clinically acceptable threshold, as indicated in the literature [33], the observed reductions may be considered acceptable in the context of baseline values ranging from 350 to 450 MPa, which are characteristic of many currently used and newly developed composites [70,79,80]. Nevertheless, the values of the recorded reductions indicate the need for caution when considering materials with an initial compressive strength of approximately 300 MPa or lower, which are registered for numerous commercially available composites [81,82,83,84]. Of particular concern is the finding that, for most of the tested solutions, the greatest changes occurred after the shortest exposure time. This suggests that early interactions may already lead to deterioration of mechanical properties not only at the surface level but also at the macro scale. A statistically significant correlation was also observed between changes in hardness and changes in compressive strength (*p* = 0.001; r = 0.5), indicating a moderate positive relationship between these parameters.

In this study, color changes were analyzed using the CIELab system. Although the CIEDE2000 (ΔE_00_) formula has increasingly been used in recent dental research due to its improved correlation with human visual perception and its ability to better reflect perceptibility and acceptability thresholds of color differences [85,86], the CIELAB system still offers important advantages for laboratory experimental studies. The CIELAB color space describes color using three coordinates directly describing changes in lightness, red–green, and yellow–blue axes, allowing not only the assessment of overall color change but also a clear, detailed analysis of variations in individual color components. This was particularly relevant in the present study. The ΔE_00_ coordinates are mathematically more complex in this regard and less intuitive to interpret. Moreover, previous studies have also demonstrated a very strong correlation between ΔE*_ab_ and ΔE_00_ values (r ≈ 0.98–0.99) [87], indicating that both formulas can be successfully used to evaluate color changes in dental materials. Importantly, despite the increasing use of ΔE_00_, the classical ΔE*_ab_ calculation remains widely applied in studies on discoloration and color stability of dental materials, which facilitates comparison with previously published research.

The ability to relatively easily obtain a wide spectrum of shades determines the high aesthetic value of dental composites, and color stability remains one of their most important functional properties. In a review article, Sampaio et al. [88] concluded that most studies indicate that mouthwashes do not induce clinically unacceptable color changes in composite materials. The evaluation of discoloration was based on the interpretation of color differences between teeth and tooth-colored restorative materials, adopting the acceptability threshold (AT) defined as the smallest color difference that is perceptible and clinically acceptable (ΔE* ≤ 2.7) [88,89]. An additional criterion was the perceptibility threshold (PT), defined as the smallest color difference detectable by an observer (ΔE* > 1.2). According to this interpretation, ΔE* values between 1.2 and 2.7 are perceptible but still aesthetically acceptable. Under this approach, none of the mouthwashes analyzed in the present study produced unacceptable color changes.

However, this interpretation constitutes a simplification. The data on perceptibility and acceptability thresholds reported in the study forming the basis for this interpretation [86] reveal considerable variability, both between different observer groups (e.g., dentists, students, technicians, dental staff) and within these groups. The confidence interval for AT at ΔE* = 2.7 ranged from 1.96 to 3.37, and for PT at ΔE* = 1.2 from 0.45 to 1.89. This broader interpretation corresponds with the traditionally accepted classification of color differences, according to which ΔE* < 1 is considered imperceptible, values from 1 to 2 are perceptible to experienced observers, and values from 2.0 are perceptible even to inexperienced observers [90]. This suggests that observers with heightened aesthetic sensitivity might consider discolorations induced in the present study by two out of twelve tested mouthwashes as unacceptable, while six mouthwashes produced perceptible changes.

An extension of interpretation was proposed by Lindsey et al. [91], who demonstrated that ΔE* values of approximately 1 resulting from changes in the L* or a* coordinates, and 2.6 for the b* coordinate of the CIELAB color space, produced perceptible and unacceptable differences. In this context, four mouthwashes (including both ethanol-containing formulations) would be considered to cause unacceptable darkening of the composite, and one mouthwash would induce a shift toward green. The recorded changes along the yellow–blue axis (b*) would remain imperceptible unless accompanied by alterations in other color coordinates.

No clear relationship was observed between specific mouthwash components and ΔE* values. For example, one mouthwash containing activated charcoal caused significant discoloration, whereas another did not; one formulation containing a blue dye (Oral B Pro Expert) produced marked color change, while another did not; conversely, one mouthwash without added colorants (KIN B5) produced some of the greatest discolorations. The presence of ethanol was not associated with consistently greater color changes, although some studies have suggested such an effect [30]. Mouthwashes containing chlorhexidine produced relatively minor discoloration, which contrasts with numerous previous reports [67,92,93], but is consistent with expectations and may be explained by the inclusion of anti-discoloration systems (ADS) [94,95]. The ADS present in both products, containing sodium metabisulfite and ascorbic acid, reduce chlorhexidine-induced staining by interfering with the chemical pathways responsible for pigment formation, including Maillard reactions and metal sulfide formation. Their mechanism of action is attributed to the reducing and antioxidant properties of these components, which limit the formation of melanoidins and metal-containing complexes capable of adsorbing onto restorative surfaces [95,96].

No statistically significant correlation was observed between ΔE* changes and hardness alterations (*p* = 0.13; r = 0.25), although such a relationship had been suggested in a previous study based on a substantially smaller number of mouthwashes [97].

After storage in certain mouthwashes, both ΔE* values and their individual components were observed to alternately increase and decrease. This suggests that the recorded color changes may result from complex interactions between the mouthwashes and the composite surfaces, involving not only matrix staining but also subsequent removal of formed discolorations or interactions with intrinsic composite pigments [98,99,100]. Therefore, it should be concluded that both the magnitude and the progression of color changes are inherently complex and multifactorial, dependent on the specific mouthwash used, and can only be fully determined through experimental investigation. This complexity also helps to explain the variability observed in recent studies, which include reports indicating the possibility of clinically unacceptable color changes [30,67,92,101] as well as studies reporting contrasting results [67,102,103]. The observed discoloration of resin composites can be attributed to several underlying mechanisms. The sorption and penetration of liquids, including ethanol, may play a significant role, as ethanol can increase the solubility of the polymer matrix and facilitate the diffusion of pigments into the material [104], particularly in composites containing hydrophilic monomers. This effect may be further enhanced by other constituents of mouthwashes. Additionally, chemical degradation of the resin matrix, including hydrolytic processes, as well as degradation of the filler–matrix interface, can lead to morphological changes such as microcrack formation, and increased roughness, which promote the adsorption and retention of chromogens, thereby contributing to staining [105,106]. Simultaneously, changes at the filler–matrix interface and within the internal structure of the material may affect its optical properties. In particular, modifications in refractive index mismatch and absorption characteristics can influence light scattering and translucency [107,108], which are critical parameters governing the aesthetic appearance of dental materials [109,110].

An important limitation of the present study arises from the simplifications inherent in conducting experiments under laboratory conditions, which—due to temporal and technical constraints—remain considerably removed from the clinical setting. One such simplification concerns the calculation of the cumulative contact time between mouthwashes and composite materials. It was assumed that the entire volume of the mouthwash is removed from the oral cavity upon spit out, and that its interaction with restorations ceases at that moment. This assumption does not reflect the clinical reality. However, it is currently not possible to determine the duration or intensity of any residual effects exerted by mouthwashes after completion of the rinsing cycle, for example due to their presence in saliva or in anatomically hard-to-reach areas. It may therefore be inferred that the adopted experimental cycles in fact simulate a shorter effective exposure time than would occur in vivo. Another simplification lies in the fact that during the 24 h experimental cycles, the mouthwashes remained in continuous contact with the specimens, which does not correspond to clinical conditions. On the other hand, this approach creates relatively more stringent experimental conditions and significantly reduces the overall duration of the study.

In clinical conditions, numerous additional factors may influence the interactions between resin composites and mouthrinses. The surface of a composite restoration is rapidly covered by an acquired pellicle composed of salivary proteins [111]. Studies have shown that the salivary pellicle can attenuate the effects of chemical agents, for example by reducing their antibacterial activity [112]; therefore, the absence of this layer in in vitro studies may lead to an overestimation of the effects of mouthrinses. On the other hand, mouthrinses are typically used immediately after toothbrushing, which may limit the clinical relevance of this protective effect. Other hand, previous studies suggest that mouthwashes, especially those containing fluoride compounds, may deposit residues on dental material surfaces, potentially altering their properties [113].

The introduction of a mouthrinse into the oral cavity is also associated with a sudden temperature drop, which may induce thermal stresses at the matrix–filler interface, promote hydrothermal degradation, and contribute to the formation of microcracks. Collectively, these phenomena may affect the mechanical properties, surface characteristics, and color stability of composite materials, acting synergistically with ongoing chemical degradation processes [114].

Moreover, the present model does not account for the synergistic effects of toothbrushing and mouthwashes. Toothbrushing initiates structural alterations of the composite surface, thereby facilitating the penetration of mouthrinse components into the compromised resin matrix. This may lead to matrix plasticization and hydrolysis of ester bonds, ultimately contributing to increased surface roughness [66,115,116] and, in some cases, to a reduction in microhardness [117], although this effect is not consistently observed [66].

It should also be noted that both water and artificial saliva are known to induce changes in the physicochemical and mechanical properties of resin-based composites. Consequently, future investigations should include testing of specimens preconditioned in these media for a period sufficient to achieve property stabilization. This consideration is particularly relevant because both previous investigations and the present study were conducted on “fresh” specimens. An open question remains as to whether, after several weeks or months, once materials have reached a stable state in an aqueous environment, they would exhibit further deterioration in mechanical or aesthetic performance when exposed to additional factors such as mouthwashes.

It must also be emphasized that the use of water as a reference medium in this study was not intended to correspond to the assumed simplified service life. If the simulated exposure time to mouthwashes is considered equivalent to six years, this assumption does not apply to the aqueous environment, since composite restorations remain in virtually continuous contact with saliva over such a period. Therefore, the use of distilled water as a control medium in the present experiment should be interpreted solely as a means of comparing the effects of a relatively non-aggressive medium under standardized conditions of temperature and humidity. It does not constitute a simulation of salivary exposure over an equivalent six-year period.

Additional limitations include the restricted number of mouthwashes evaluated and the selection of a single composite material. A nanocomposite was chosen for testing in light of evidence suggesting that this category of materials demonstrates greater stability of mechanical properties and color when exposed to mouthwashes [46,103,118]. Accordingly, more pronounced changes might be anticipated if microhybrid composites or other material classes were investigated. Easy Fill Nano Composite was selected as an investigation of contemporary nano-/nanohybrid composites. Its resin matrix consists of UDMA and bis-GMA combined with a diluent monomer, and it contains inorganic fillers of mixed nano- and micro-sized particles. All of the matrix components in this material are also used in other commercially available resin composites [31,32,119,120], and identical monomer combinations have also been reported [31]. Nevertheless, the limited independent validation of this specific material restricts generalizability. Therefore, the results should be considered exploratory, highlighting potential trends and mechanisms in mouthwash–composite interactions rather than definitive effects for all resin composites. Future studies including multiple well-characterized materials are warranted.

Another limitation of the present study is that flexural strength testing, although standardized by ISO 4049 [36], was not performed. Instead, tensile- and compressive-related properties were evaluated separately using DTS and compressive strength, which provide complementary insight into the material’s mechanical behavior. Inclusion of flexural strength could further enhance comparability with other studies and should be considered in future research.

## 5. Conclusions

What is particularly important, mouthwashes did not affect the DTS of the composite material used for direct restorations; nevertheless, they did have a significant impact on its CS and microhardness. The loss of mechanical performance was typically most pronounced during the initial period of exposure to mouthwashes, which should be regarded as clinically unfavorable in view of the continuous tribological processes accompanying the functional service of restorations in the oral environment. The presence of ethanol in the chemical composition of mouthwashes was confirmed as a factor increasing the risk of substantial alterations in composite properties. Although mouthwashes containing ethyl alcohol demonstrated a marked impact on the mechanical properties of composite materials, the results do not indicate that their effects are inherently more deleterious than those of many ethanol-free formulations. The frequently suggested correlation between changes in composite hardness and the pH values of mouthwashes was not confirmed.

The use of mouthwashes was also associated with discoloration of composite materials. No consistent pattern was identified in this respect, suggesting that the extent of staining depends primarily on the specific formulation of the mouthwash. Preparations containing chlorhexidine in combination with an Anti-Discoloration System (ADS) likewise did not cause unacceptable discoloration. Considering typical thresholds of perception, the recorded changes were noticeable but remained within clinically acceptable limits from an aesthetic standpoint.

## Figures and Tables

**Table 1:** Chemical composition and pH values of the media used during the experiment.

Medium	Manufacturer	pH Value	Components Important for Categorization	Composition
Water	-	5.8	-	-
Beverly Hills perfect white black	Purity Laboratories Ltd., Dublin, Ireland	5.5	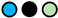	Aqua, Alcohol, Glycerin, PEG-40 Hydrogenated Castor Oil, Sodium Lauryl Sulfate, Sodium Saccharin, Aroma, Sodium Benzoate, Charcoal Powder, Sodium Fluoride, dyes (CI47005, CI44090)
Listerine Fresh Burst	Johnson & Johnson Poland Ltd., Warszawa, Poland	4.18	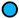	Water, alcohol, sorbitol, poloxamer 407, benzoic acid, sodium saccharin, eucalyptol, methyl salicylate, flavor, thymol, menthol, sodium benzoate, dyes (CI47005, CI42053).
Listerine Advanced White	Johnson & Johnson Poland Ltd., Warszawa, Poland	6.13	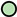	Water, sorbitol, propylene glycol, tetrapotassium pyrophosphate, pentasodium triphosphate, citric acid, poloxamer 407, sodium methyl cocoyl taurate, caprylyl glycol, eucalyptol, thymol, sodium saccharin, menthol, sodium fluoride (220 ppm F), sucralose.
Dentica Black White	Torf Corporation Ltd., Kąty Wrocławskie, Poland	5.19	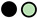	Water, Glycerin, PEG-40, Hydrogenated Castor Oil, Charcoal Powder, Sodium Fluoride, Tetrapotassium Pyrophosphate, Tetrasodium Pyrophosphate, Aroma, Polyglyceryl-10 Myristate, Polyglycerin-10, Sodium Dehydroacetate, Sodium Citrate, Citric Acid, Sodium Benzoate, Limonene, dyes (CI42051, CI16255, CI47005).
Colgate Plax	Colgate-Palmolive Poland Ltd., Warszawa, Poland	5.8	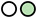	Water, Glycerin, Propylene Glycol, Sorbitol, Poloxamer 407, Aroma, Cetylpyridinium Chloride, Potassium Sorbate, Sodium Fluoride (0.05%-225 ppm F), Sodium Saccharin, Menthol, dye CI 42051.
Biomed Citrus Fresh	Splat Global LLC, Riga, Latvia	6.56	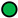	Aqua, Hydrogenated Starch Hydrolysate, Calcium Lactate, Betula Alba Leaf Extract, Plantago Lanceolata Leaf Extract, Sodium Lauroyl Sarcosinate, Menthol, Stevia Rebaudiana Leaf Extract, Sodium Chloride, Ananas Sativus Fruit Extract, Arginine, Citrus Limon Peel Oil, Magnesium Aspartate, Zinc Gluconate, Copper Gluconate, Aroma, Maltodextrin, Glycerin, Tetrasodium Glutamate Diacetate, Polyglyceryl-4 Laurate/Sebacate, Polyglyceryl-6 Caprylate/Caprate, Benzyl Alcohol, Sodium Benzoate, Potassium Sorbate, Citric Acid, Limonene, Eugenol.
Biomed Super White	Splat Global LLC, Riga, Latvia	5.76	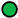	Water, Hydrogenated Starch Hydrolysate, Sodium Coco-Sulfate, Betula Alba Leaf Extract, Plantago Lanceolata Leaf Extract, Cocos Nucifera Fruit Extract, Lactobacillus, Xylitol, Stavia Rebaudiana Leaf Extract, Menthol, Papain, Polyglyceryl-4 Laurate/Sebacate, Polyglyceryl-6 Caprylate/Caprate, Aroma, Arginine, Magnesium Aspartate, Zinc Gluconate, Copper Gluconate, Glycerin, Benzyl Alcohol, Sodium Benzoate, Potassium Sorbate, Citric Acid, Tetrasodium Glutamate Diacetate.
ORAL-B Pro-Expert	Procter and Gamble DS Poland Ltd., Warszawa, Poland	4.19	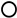	Water, Glycerin, Aroma, Cetylpyridinium Chloride, Poloxamer 407, Methylparaben, Sodium Saccharin, Cinnamal, Propylparaben, Eugenol, dye CI 42090.
KIN B5	Laboratorios KIN, Barcelona, Spain	5.96	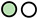	Water, Sorbitol, Glycerin, PEG-40 Hydrogenated Castor Oil, Propylene Glycol, Xylitol, Panthenol, Zinc Lactate, Sodium Methylparaben, Citric Acid, Fragrance, Disodium EDTA, Niacinamide, Cetylpyridinium Chloride, Menthol, Sodium Fluoride, Propylparaben, d-Limonene.
Curasept ADS 205	Curasept S.p.A., Saronno, Italy	5.25	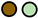	Water, xylitol, propylene glycol, PEG-40 hydrogenated castor oil, ascorbic acid, chlorhexidine digluconate, flavor, sodium fluoride, poloxamer 407, sodium benzoate, sodium metabisulfite, sodium citrate, dye CI 42090.
Curasept ADS 012	Curasept S.p.A., Saronno, Italy	5.15	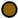	Water, Xylitol, Propylene Glycol, PEG-40, Hydrogenated Castor Oil, Ascorbic Acid, Chlorhexidine Digluconate, Fragrance, Poloxamer 407, Sodium Methyldisulfonate, Sodium Citrate, dye CI 42090.
Curasept Biosmalto Sensitive Teeth	Curasept S.p.A., Saronno, Italy	5.49	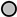	Water, Xylitol, Fluorohydroxyapatite, Mg-Sr-Carbonate Hydroxyapatite conjugated with Chitosan, Strontium Acetate, Cocamidopropyl Betaine, Poloxamer 407, Potassium Acesulfame, Xanthan Gum, Ammonium Acryloyldimethyltaurate/VP Copolymer, Chlorphenesin, Ethylhexylglycerin, Phenoxyethanol, Sodium Benzoate, Sodium Monofluorophosphate, Aroma, Methylisothiazolinone, Citric Acid.

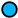
—ethanol; 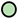
—fluorides; 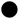
—charcoal; 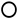
—cetylpyridinium chloride; 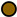
—chlorhexidine; 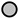
—apatites; 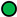
—based on natural ingredients.

## Data Availability

The original contributions presented in this study are included in the article. Further inquiries can be directed to the corresponding author.

## Abbreviations

The following abbreviations are used in this manuscript:

CS	Compressive Strength
DTS	Diametral Tensile Strength
HV	Vickers Hardness
ADS	Anti-Discoloration System
ΔE*	Color difference
L*	Lightness coordinate
a*	coordinate red–green
b*	coordinate yellow–blue
AT	acceptability threshold
PT	perceptibility threshold
ANOVA	one-way analysis of variance
